# Continuance intention toward AI-driven mHealth apps: an extended expectation confirmation model based on a cross-sectional study of young Chinese adults

**DOI:** 10.3389/fpubh.2026.1946882

**Published:** 2026-10-08

**Authors:** Jiaqi Wang, Donghwa Chung

**Affiliations:** 1 School of Journalism and Communication, Xiamen University, Xiamen, China; 2 School of Journalism and Communication, Central China Normal University, Wuhan, China

**Keywords:** artificial intelligence, continuance intention, mobile health apps, survey, young adults

## Abstract

**Background:**

Cutting-edge artificial intelligence (AI) technologies have accelerated the global development of AI-driven mHealth apps, offering diverse functionalities to support personal health management. While China’s AI health industry has expanded rapidly within a collectivist cultural context, limited empirical attention has been given to how specific AI features are associated with users’ continued use of these apps, particularly among young adults.

**Objective:**

Guided by the Expectation Confirmation Model and extended AI-related features, this study examined the factors associated with continuance intention toward AI-driven mHealth apps among young Chinese adults aged 18–34.

**Methods:**

A cross-sectional online survey was conducted, collecting data from 319 valid respondents. Data were analyzed using SPSS 29.0 and Jamovi 2.6.24 to evaluate direct, mediation, and serial mediation associations among the constructs.

**Results:**

The findings demonstrate that satisfaction statistically bridged the indirect associations between core ECM constructs (expectation confirmation and perceived performance) and continuance intention, as well as between AI quality and continuance intention. Furthermore, bootstrapping analysis supported a sequential mediation pathway, showing that expectation confirmation was indirectly associated with continuance intention through the proposed sequential pathway of perceived performance and satisfaction.

**Conclusion:**

The findings suggest that developers should consider the technical reliability and performance of AI-driven health management and monitoring functions to better align with user needs. Moreover, collaboration between health experts and industry practitioners may support the development of content for AI-driven mHealth adoption.

## 1 Introduction

Mobile health (mHealth) apps, defined as smartphone-based software designed to support health care services, outcomes, and research, have expanded rapidly worldwide in recent years (1). These apps support disease prevention and health management through functions such as remote monitoring, medication reminders, and health education (2–4). Their utilization has been documented across various contexts, including the United States, Europe, and East Asia (5–7). A recent report indicates that, by 2019, the number of mHealth application users in China had reached approximately 466 million, reflecting the widespread development and adoption of these digital health platforms in the country, with users actively engaging in a range of health-related services and functions (8, 9). This expanding user base has increased academic and industry interest in Chinese users’ needs and experiences with mHealth apps.

Advances in artificial intelligence have accelerated the global development of AI-driven mHealth apps. These apps use artificial intelligence to process health data, provide interactive assistance, and offer tailored health guidance (10). Their functions include chronic disease prediction, personalized services, and medical error prevention (11–13). Against this background, recent industry examples suggest increasing user engagement with AI-driven mHealth apps in China. For instance, Ping An Good Doctor was one of the leading mHealth apps, reportedly reaching 16.2 million registered users in 2020 (8). Similarly, iFlytek Xiaoyi has reportedly accumulated over 20 million users and handles more than 300,000 daily health-related inquiries in China (14). Furthermore, AI-driven mHealth apps encompass diverse categories and service models, with functions and service offerings varying across platforms. For instance, JD Health, Alibaba Health, and WeDoctor provide distinct combinations of online consultations, medication services, and digital health management (15). This diversity reflects the broader landscape of health platforms that integrate multiple health-related services within distinct service models (15).

Investigations of Chinese heavier users of AI-driven mHealth apps demonstrated that, compared with lighter users, they tend to exhibit higher digital literacy and greater confidence and actively engage with these platforms to seek support and inquire about health-related questions (16). Additionally, frequent users in China generally reported greater trust in these platforms, particularly satisfaction with their interaction experiences and the helpfulness of these chatbots (17). Taken together, recent descriptive studies provide valuable insights into Chinese users’ engagement with and perceptions of AI-driven mHealth apps. However, they provide limited insight into the underlying associations related to users’ continuance intention, warranting further investigation.

With the growing body of research on users’ adoption intentions toward mHealth apps worldwide, established technology acceptance theories, particularly the Technology Acceptance Model (TAM) and the Unified Theory of Acceptance and Use of Technology (UTAUT), have provided important theoretical foundations for explaining users’ acceptance of digital health technologies (18–21). While TAM and UTAUT have received considerable attention and use in technology research, these theories primarily focus on initial adoption (22), whereas the Expectation Confirmation Model (ECM) stands out as providing a more direct theoretical basis for examining continuance intention based on users’ prior use experiences (22, 23). Accordingly, ECM provides a suitable theoretical lens for examining continuance intention toward AI-driven mHealth apps.

In this context, growing empirical research has examined users’ intentions toward AI-driven mHealth apps (24, 25). However, limited attention has been paid to how AI-related features shape continuance intention, particularly in the Chinese context. In addition, existing mHealth research has also primarily focused on older adults or patients with specific diseases, such as diabetes and epilepsy (26–29). Consequently, younger populations remain relatively understudied despite increasing health risks and engagement in preventive health behaviors (30–32).

These gaps are particularly noteworthy considering that existing ECM-based mHealth research has generally focused on conventional factors underlying continuance intention (33, 34), leaving limited understanding of how AI-specific features may be incorporated into the continuance process. Examining these associations through ECM can therefore provide insight into the explanatory value of the model in the emerging AI-driven mHealth context, particularly among young Chinese adults, an understudied population. Guided by ECM, this study investigates the associations between psychological factors, AI-related features, and continuance intention toward AI-driven mHealth apps among young Chinese adults aged 18–34. The findings contribute to the application of ECM by incorporating AI-related features into the examination of continuance intention, while providing practical implications for supporting sustained app use and preventive health engagement.

## 2 Literature review

### 2.1 Expectation confirmation model (ECM)

The ECM is derived from expectation-confirmation theory and explains the cognitive evaluation process underlying continuance intention, emphasizing expectation confirmation, perceived performance, and satisfaction (35). Following prior research (35, 36), this study defines expectation confirmation as users’ judgment of whether AI-driven mHealth apps meet their initial expectations. Perceived performance refers to users’ evaluations of the characteristics, advantages, and outcomes of these apps. Satisfaction denotes users’ overall psychological response to the gap between expected and experienced performance, while continuance intention refers to young Chinese adults’ intention to keep using AI-driven mHealth apps.

Expectation confirmation model has been widely applied in technology-related contexts, including mobile commerce, online learning, and digital health (37–39). Recent studies have also extended the model to AI-mediated contexts by incorporating AI-related factors, such as perceived intelligence (40), perceived anthropomorphism (22), and visual attractiveness (41). Extending this line of research, the present study applies ECM and incorporates two AI-related features, perceived anthropomorphism and AI quality, to examine factors associated with young Chinese adults’ continuance intention toward AI-driven mHealth apps.

### 2.2 ECM predictors on satisfaction

In the ECM framework, expectation confirmation is established as a key construct associated with satisfaction (35). Studies in digital media and technology contexts have repeatedly reported a positive association between expectation confirmation and user satisfaction. In digital health research, this positive association has also been observed across online healthcare platforms, fitness and health apps, and health science popularization videos (42–44). Perceived performance has also been examined as a construct associated with satisfaction in the ECM framework. A prior study reported a positive association between perceived usefulness and satisfaction, and perceived usefulness has often been conceptualized as closely related to perceived performance (45). Empirical studies in digital health contexts have likewise reported positive associations between perceived performance and user satisfaction (42, 46, 47). Based on this theoretical and empirical basis, expectation confirmation and perceived performance are expected to be positively associated with satisfaction with AI-driven mHealth apps among young Chinese adults. Therefore, this study proposes the following hypotheses:

*Hypothesis 1a*: Expectation confirmation is positively associated with young Chinese adults’ user satisfaction.

*Hypothesis 1b*: Perceived performance is positively associated with young Chinese adults’ user satisfaction.

### 2.3 Direct effects of AI-related features on satisfaction

While ECM suggests that satisfaction is primarily shaped by users’ evaluations of confirmation and perceived performance, AI-related features may also be directly associated with satisfaction rather than necessarily being positioned as antecedents of these cognitive evaluations. According to the Computers Are Social Actors (CASA) theory, anthropomorphic factors, or human-like characteristics, tend to shape users’ social experiences and responses toward AI applications (48). Building on this theoretical perspective, prior studies have found direct associations between AI-related features and satisfaction with intelligent customer service bots. While Xie et al. (49) reported indirect associations between anthropomorphic cues and interaction satisfaction, recent evidence provides further empirical support that anthropomorphic design is directly associated with satisfaction (50), indicating that AI-related features can be directly associated with users’ satisfaction without necessarily operating through prior cognitive evaluations. Thus, these theoretical and empirical perspectives provide a basis for the current study to treat AI-related features as parallel cues of satisfaction, rather than positioning them as antecedents of ECM evaluations.

Anthropomorphism has been defined in prior studies as the attribution of human characteristics to non-human entities, including human-like emotions, mental states, and behaviors (22, 51). In the context of AI, anthropomorphism refers to the extent to which users perceive AI systems as possessing human-like characteristics, such as emotional expression, responsiveness, and contingent interactivity (52, 53). In this study, perceived anthropomorphism refers to users’ perceptions that AI-driven mHealth apps possess human-like features during their interactions with the apps. Previous research has reported an association between perceived anthropomorphism and more positive emotional responses toward AI-based systems (50, 54). In related digital service and human–AI interaction contexts, perceived anthropomorphism has also been positively associated with user satisfaction. For instance, users who perceive AI apps as more human-like (also known as perceived anthropomorphism) reported higher levels of satisfaction with the interaction experience (55, 56). Similarly, research on humanoid service robots in hospitality contexts suggests that anthropomorphic features are positively associated with user satisfaction (57). Taken together, these findings suggest a positive association between perceived anthropomorphism and user satisfaction in AI-driven mHealth apps contexts.

*Hypothesis 2a*: Perceived anthropomorphism is positively associated with young Chinese adults’ user satisfaction.

Perceived quality has been defined as an individual’s subjective assessment of the overall excellence or superiority of a product or service (58). More recently, this perspective has been applied to AI-driven platforms, with Dantsoho et al. (59) characterizing AI quality in terms of users’ perceptions of the overall quality of AI-powered e-banking services. Drawing on this conceptualization, the present study defines AI quality as users’ holistic perception of the quality of AI-driven mHealth apps.

Previous studies have demonstrated positive associations between perceived quality and user satisfaction across various digital service contexts. For instance, prior research has demonstrated that perceived quality is positively associated with consumer satisfaction within online-to-offline commerce (60). Similarly, Gu (61) found a positive relationship between overall service quality and customer satisfaction among tourists. In AI-enabled service contexts, Dantsoho et al. (59) reported a positive association between AI quality and user satisfaction in AI-powered e-banking services. Taken together, these findings suggest that AI quality may be positively associated with user satisfaction in the context of AI-driven mHealth apps. Therefore, the following hypothesis is proposed:

*Hypothesis 2b*: AI quality is positively associated with young Chinese adults’ user satisfaction.

### 2.4 Mediating mechanisms linking satisfaction and continuance intention

Prior literature has identified expectation confirmation as an important factor associated with user satisfaction (42–44). Perceived performance has also been positively associated with user satisfaction (42, 46, 47). Moreover, recent studies suggest a positive association between perceived anthropomorphism and user satisfaction in AI-related contexts (55, 56). Likewise, perceived quality has also shown a positive association with user satisfaction (59–61).

Previous research has shown positive associations between various antecedents (ECM constructs and AI-related features) and user satisfaction. Beyond these direct associations, increasing attention has also been directed toward the potential outcomes of satisfaction. For instance, user satisfaction has been positively associated with continuance intention to use mHealth apps (35, 62, 63). Similarly, users with higher satisfaction toward mobile health services (e.g., service features and interface design) tend to exhibit stronger continuance intention over time (35). In other words, greater satisfaction with the functions and design of mobile health services is associated with stronger continuance intention toward such apps. Accordingly, the present study further proposes that user satisfaction is positively associated with continuance intention toward AI-driven mHealth apps. Taken together, this study proposes that satisfaction mediates the relationships between ECM constructs, AI-related features, and young Chinese adults’ continuance intention toward AI-driven mHealth apps (hereafter, continuance intention). Therefore, the following hypotheses are proposed:

*Hypothesis 3a*: Satisfaction mediates the association between expectation confirmation and continuance intention among young Chinese adults.

*Hypothesis 3b*: Satisfaction mediates the association between perceived performance and continuance intention among young Chinese adults.

*Hypothesis 4a*: Satisfaction mediates the association between perceived anthropomorphism and continuance intention among young Chinese adults.

*Hypothesis 4b*: Satisfaction mediates the association between AI quality and continuance intention among young Chinese adults.

### 2.5 The serial mediating role of perceived performance and satisfaction

According to the ECM, expectation confirmation tends to be positively associated with individuals’ perceived usefulness (i.e., perceived performance), whereas disconfirmation is likely to be negatively associated with such perceptions (35). Consistent with this proposition, empirical studies find that users with higher levels of expectation confirmation tend to exhibit higher perceived performance (41, 64). Moreover, scholars report a positive association between perceived performance and satisfaction (42, 47). In addition, satisfaction positively relates to continuance intention (35, 62). Based on these empirical findings, it is plausible that perceived performance and satisfaction may serially mediate the association between expectation confirmation and continuance intention, with the pathway as follows: expectation confirmation → perceived performance → satisfaction → continuance intention. Thus, the following hypothesis is proposed:

*Hypothesis 5*: The association between expectation confirmation and continuance intention is sequentially mediated by perceived performance and satisfaction among young Chinese adults.

The hypothesized model is presented in Figure 1, which outlines the proposed relationships among the key variables.

**Figure 1:**
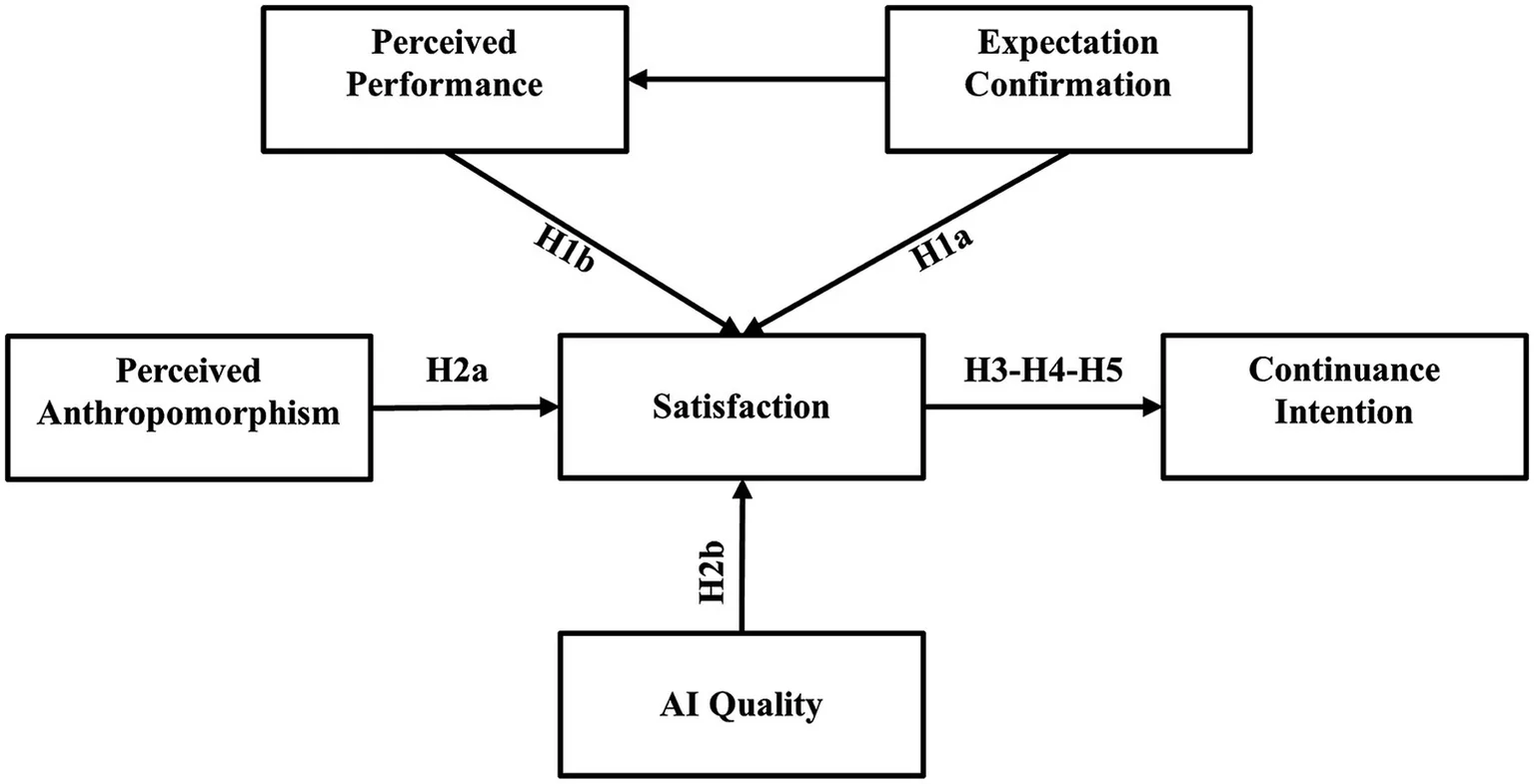
Hypothesized model of the antecedents of young Chinese adults’ continuance intention toward AI-driven mHealth apps.

## 3 Methods

### 3.1 Data collection and ethics

Non-probability sampling was employed. Although this approach limits population generalizability, it is widely accepted for theory-driven hypothesis testing in the health communication field (65). Participants were recruited from Credamo (www.credamo.com; accessed January 10, 2026), a widely used Chinese online survey platform that has been extensively applied in behavioral research in China (66). Data were collected from January 10 to June 5, 2026, using rolling recruitment to obtain a sufficient number of eligible responses. To be eligible, respondents were required to (1) be aged 18–34 years; (2) have used an mHealth application (e.g., hospital diagnostic services, health monitoring tools, or AI-based health chatbots) within the past 3 months; (3) demonstrate awareness of AI-enabled mHealth concepts through screening questions; and (4) have experience using AI-enabled mHealth services for purposes such as obtaining health information, participating in health consultations, or managing health conditions. Respondents who did not meet the eligibility criteria were excluded from the final sample.

Ethical approval for this study was obtained from the Ethics Committee of the School of Journalism and Communication, Central China Normal University (Approval number: CCNUIRB20260102; approved on January 2, 2026). An informed consent form was presented to all respondents on the first page of the online survey. Only respondents who voluntarily agreed to participate after being fully informed about the purpose and procedures of the study were included. The informed consent form also emphasized that participation was voluntary and that respondents had the right to withdraw from the study at any time without penalty.

Sample size estimation procedures based on an *a priori* power analysis. First, following the 10-times rule of thumb (67), the maximum number of predictors for any endogenous variable was four, indicating a minimum required sample size of 40. Second, an *a priori* power analysis was conducted using G*Power 3.1.9.4 (68). Assuming four predictors, a medium effect size (*f*^2^ = 0.15), α = 0.05, and statistical power of 0.95, the minimum required sample size was 129. The final valid sample consisted of 319 respondents, exceeding both thresholds. Therefore, the sample size was considered sufficient for the present analysis.

### 3.2 Measurements

In this study, AI-driven mHealth apps were treated as a broader class of technologies because the aim was to investigate users’ experiences with AI-mediated health interactions in general, rather than to compare the characteristics or performance of particular applications. All structured scales employed in this study were adapted from prior research and were originally developed in English. To ensure consistency with the original item meanings, a back-translation procedure was conducted, in which the translated items were compared against the original versions (69). The translated instrument was subsequently reviewed and refined by two experts in linguistics and translation at Central China Normal University. Content validity was assessed by three scholars in the field of health communication, who evaluated the relevance, comprehensiveness, and representativeness of each construct (70). Based on their feedback, necessary revisions were carefully implemented.

All measures in this study were assessed using a five-point Likert scale ranging from “strongly disagree” to “strongly agree.” Perceived anthropomorphism was measured with four items adapted from prior research (71). Participants were asked to indicate their agreement with statements regarding the perceived human-like features of AI-driven mHealth apps during interaction. Sample items included “Using AI-driven mHealth apps feels similar to interacting with a real person” and “The AI-driven mHealth apps feel friendly” (*M* = 2.65, SD = 1.25, *α* = 0.94).

The perceived performance scale was adapted from Alnaser et al. (41) to assess participants’ perceptions of the characteristics, advantages, and outcomes of AI-driven mHealth apps. The scale comprised three items. Sample items included “AI-driven mHealth apps help me use medication correctly” and “AI-driven mHealth apps help me effectively obtain health-related knowledge” (*M* = 4.19, SD = 0.59, *α* = 0.75).

AI quality was operationalized using four items adapted from Pol (72), who adopted the perceived quality measure originally developed in a prior study (58). In the current study, the subject was changed to AI-driven mHealth apps, and AI quality was assessed as a single reflective construct. A sample item was “The likely quality of AI-driven mHealth apps is extremely high.” (*M* = 3.98, SD = 0.68, *α* = 0.79).

The expectation confirmation measure was adapted from an existing measure (41) and consisted of three items assessing the extent to which participants’ initial expectations of AI-driven mHealth apps were met. Example items were “My experience with AI-driven mHealth apps is better than I expected” and “The health management benefits of AI-driven mHealth apps are better than I expected” (*M* = 4.28, SD = 0.64, *α* = 0.80).

Satisfaction was measured using two items adapted from a prior study (22), assessing participants’ overall satisfaction with AI-driven mHealth apps. Example items were “I am satisfied with the services provided by AI-driven mHealth apps” and “I think the decision to use AI-driven mHealth apps is wise” (*M* = 4.20, SD = 0.60, *α* = 0.73).

Continuance intention was measured using two items adapted from Yu et al. (73). Participants indicated the extent to which they agreed with each item. Example items were “I intend to continue using AI-driven mHealth apps and not stop using them” and “If I can, I will use AI-driven mHealth apps as much as possible” (*M* = 4.14, SD = 0.73, *α* = 0.80).

### 3.3 Reliability and validity tests

Prior to hypothesis testing, the reliability and validity of the measurement instruments were assessed to ensure construct stability, internal consistency, and measurement accuracy. Composite reliability (CR) and average variance extracted (AVE) were evaluated, with the results reported in Table 1. All CR values exceeded the recommended threshold of 0.70, indicating satisfactory internal consistency. In addition, all AVE values were above 0.50, supporting adequate convergent validity (74). Cronbach’s alpha coefficients ranged from 0.73 to 0.94, exceeding the recommended threshold of 0.70, thereby confirming acceptable reliability across all constructs.

**Table 1:** Construct reliability and validity.

**Variable**	**items**	**Correlation loading**	**CA**	**CR**	**AVE**
Perceived anthropomorphism	PA1	0.91	0.94	0.95	0.82
	PA2	0.81			
	PA3	0.94			
	PA4	0.95			
Perceived performance	PP1	0.83	0.75	0.84	0.65
	PP2	0.77			
	PP3	0.81			
AI quality	AQ1	0.81	0.79	0.86	0.61
	AQ2	0.81			
	AQ3	0.74			
	AQ4	0.76			
Expectation confirmation	EC1	0.84	0.80	0.88	0.70
	EC2	0.81			
	EC3	0.86			
Satisfaction	S1	0.89	0.73	0.87	0.77
	S2	0.87			
Continuance intention	CI1	0.89	0.80	0.88	0.79
	CI2	0.89			

Prior to hypothesis testing, preliminary assessments were conducted to evaluate potential common method bias (CMB) and multicollinearity. CMB was assessed using Harman’s single-factor test (75). The results indicated that a single factor accounted for 46.04% of the total variance, which was below the commonly used threshold of 50%. However, Harman’s single-factor test provides limited evidence regarding common method variance; therefore, CMB cannot be completely ruled out. In addition, multicollinearity was examined using variance inflation factor (VIF) values, following procedures reported in prior research (76). The VIF values ranged from 1.000 to 2.271, which were below the commonly recommended threshold of 3.0 (77), indicating that multicollinearity was not a concern in the structural model.

Discriminant validity was assessed using the Fornell–Larcker criterion (78). The results showed that the square roots of the average variance extracted (AVE) values for each construct exceeded the corresponding inter-construct correlations, indicating satisfactory discriminant validity (see Table 2).

**Table 2:** Results of the Fornell–Larcker criterion.

Construct	**1**	**2**	**3**	**4**	**5**	**6**
Perceived anthropomorphism	0.90					
Perceived performance	0.05	0.80				
AI quality	0.22**	0.68**	0.78			
Expectation confirmation	0.21**	0.58**	0.61**	0.83		
Satisfaction	0.08	0.61**	0.65**	0.63**	0.88	
Continuance intention	0.15**	0.63**	0.67**	0.64**	0.65**	0.89

**p < 0.01.

Discriminant validity was further assessed using the heterotrait–monotrait (HTMT) ratio, which evaluates the distinctiveness between constructs (79). Following Henseler et al. (80), HTMT values below the recommended threshold of 0.90 indicate satisfactory discriminant validity. As shown in Table 3, all HTMT values were below this threshold, providing further support for the discriminant validity of the constructs.

**Table 3:** HTMT discriminant validity results.

Construct	**1**	**2**	**3**	**4**	**5**	**6**
Perceived anthropomorphism	–					
Perceived performance	0.10	–				
AI quality	0.25	0.86	–			
Expectation confirmation	0.24	0.76	0.78	–		
Satisfaction	0.09	0.82	0.85	0.83	–	
Continuance intention	0.20	0.84	0.87	0.83	0.87	–

Given the extended theoretical model, confirmatory factor analysis (CFA) was conducted to assess whether the measurement items adequately loaded onto their intended constructs. Following established methodological procedures, the results indicated an acceptable model fit (χ^2^/df = 2.24, CFI = 0.956, TLI = 0.944, RMSEA = 0.062, 90% CI [0.052, 0.072]), suggesting the adequacy of the measurement structure.

### 3.4 Hypothesis testing approach

In this study, the empirical analysis comprised several analytical procedures. First, descriptive analyses of demographic characteristics were conducted using SPSS 29.0. Second, CFA was performed using Jamovi 2.6.24 to evaluate the measurement model and assess its fit to the observed data. Third, the structural model was assessed to examine the hypothesized direct relationships among the study constructs. Finally, Hayes’ PROCESS macro in SPSS 29.0 was used to test the proposed mediation effects, with Model 4 examining parallel mediation and Model 6 examining serial mediation. These analyses corresponded to the measurement and structural relationships specified in the conceptual framework.

## 4 Results

### 4.1 Descriptive statistics

A total of 319 valid responses were obtained. Gender was distributed as follows: female (*N* = 168, 52.7%) and male (*N* = 151, 47.3%). The sample therefore included relatively balanced numbers of female and male participants. Education level was distributed as follows: high school (*N* = 25, 7.8%), undergraduate (*N* = 244, 76.5%), master’s degree (*N* = 42, 13.2%), and doctoral degree (*N* = 8, 2.5%). The age distribution was as follows: 18–23 years (*N* = 83, 26.0%), 24–29 years (*N* = 114, 35.7%), and 30–34 years (*N* = 122, 38.2%). Monthly income was distributed as follows: below 3,000 CNY (*N* = 22, 6.9%), 3,001–5,000 CNY (*N* = 50, 15.7%), 5,001–8,000 CNY (*N* = 78, 24.5%), 8,001–15,000 CNY (*N* = 92, 28.8%), and 15,001 CNY and above (*N* = 77, 24.1%).

### 4.2 Hypothesis testing results

Prior to hypothesis testing (H1a–b and H2a–b), the overall model fit was assessed to evaluate the adequacy of the structural model. The results indicated an acceptable model fit (χ^2^/df = 3.03, CFI = 0.981, TLI = 0.942, RMSEA = 0.080, 95% CI [0.036, 0.127], SRMR = 0.014). The current study demonstrated differences in fit indices between the CFA and pathway model. CFA assesses the measurement structure through correlations among latent constructs without specifying directional pathways, whereas the structural model incorporates the hypothesized pathways among key variables (81). Thus, differences in model specification, degrees of freedom, and parameter estimates can result in different overall fit indices. In this context, although the RMSEA (0.080) was comparatively higher than the SRMR (0.014), this discrepancy may partly reflect the incorporation of degrees of freedom in RMSEA, which can be sensitive to model complexity. Overall, the combined fit indices indicated an acceptable model fit.

The pathway model explained 53% of the variance in satisfaction (*R*^2^ = 0.53). Regarding the direct associations between ECM predictors and satisfaction, expectation confirmation was positively associated with satisfaction (*B* = 0.41, *t* = 9.00, 95% CI [0.32, 0.50], *p* < 0.001). Similarly, perceived performance was positively associated with satisfaction (*B* = 0.34, *t* = 6.90, 95% CI [0.25, 0.44], *p* < 0.001). Thus, H1a and H1b were supported. In addition, the direct associations between AI-related features and satisfaction were examined. Perceived anthropomorphism was not significantly associated with satisfaction (*B* = 0.02, *t* = 0.83, 95% CI [−0.03, 0.08], *p* > 0.05). Thus, H2a was rejected. Moreover, AI quality was positively associated with satisfaction (*B* = 0.54, *t* = 14.47, 95% CI [0.47, 0.62], *p* < 0.001). Therefore, H2b was supported.

### 4.3 Analysis of indirect associations

Mediating associations (H3a–b and H4a–b) were examined using Hayes’ PROCESS macro (Model 4) with 2,000 bootstrap resamples and bias-corrected 95% confidence intervals. Four separate mediation models were estimated, as PROCESS Model 4 can be applied repeatedly to examine the indirect association of each focal predictor (82). This approach has been used to estimate separate indirect associations for distinct predictors within the same conceptual framework. Gender, age, education level, and monthly income were included as control variables in all four mediation models, with the control variables, total associations, direct associations, and indirect associations reported in Table 4.

**Table 4:** Summary of four mediation analyses for continuance intention.

**Path/Association**	**Model 1**	**Model 2**	**Model 3**	**Model 4**
*Total effect*	0.75***	0.77***	0.07*	0.71***
95% CI	[0.66, 0.85]	[0.66, 0.88]	[0.01, 0.14]	[0.63, 0.80]
*Direct effect*	0.48***	0.46***	0.06*	0.47***
95% CI	[0.36, 0.59]	[0.34, 0.59]	[0.01, 0.11]	[0.36, 0.58]
*Mediator → DV*	0.47***	0.52***	0.78***	0.44***
*Indirect effect*				
*B*	0.28	0.31	0.02	0.24
Boot SE	0.05	0.05	0.02	0.04
95% CI	[0.18, 0.38]	[0.21, 0.42]	[−0.03, 0.06]	[0.16, 0.33]
*Controls → DV*				
Gender	−0.10	−0.06	−0.05	−0.05
Age	0.06	0.02	0.02	0.02
Education level	0.10	0.07	0.07	0.08
Monthly income	−0.03	−0.01	0.01	−0.01
Hypothesis decisions	Supported	Supported	Rejected	Supported

*p < 0.05; **p < 0.01; ***p < 0.001.

The first model (mediated pathway involving expectation confirmation) showed a significant total effect between expectation confirmation and continuance intention (*B* = 0.75, t = 14.97, 95% CI [0.66, 0.85], *p* < 0.001). The indirect association between expectation confirmation and continuance intention through satisfaction was also significant (*B* = 0.28, BootSE = 0.05, 95% CI [0.18, 0.38]), supporting H3a. In addition, the second mediation pathway showed a significant total effect between perceived performance and continuance intention (*B* = 0.77, *t* = 13.82, 95% CI [0.66, 0.88], *p* < 0.001). The indirect association between perceived performance and continuance intention through satisfaction was also significant (*B* = 0.31, BootSE = 0.05, 95% CI [0.21, 0.42]), supporting H3b. In terms of the mediated pathway involving perceived anthropomorphism (H4a), the total effect between perceived anthropomorphism and continuance intention was significant (*B* = 0.07, *t* = 2.21, 95% CI [0.01, 0.14], *p* < 0.05). However, the indirect association between perceived anthropomorphism and continuance intention through satisfaction was not significant (*B* = 0.02, BootSE = 0.02, 95% CI [−0.03, 0.06]). Lastly, the fourth mediation model demonstrated a significant total effect between perceived AI quality and continuance intention (*B* = 0.71, *t* = 15.71, 95% CI [0.63, 0.80], *p* < 0.001). The indirect association between perceived AI quality and continuance intention through satisfaction was also significant (*B* = 0.24, BootSE = 0.04, 95% CI [0.16, 0.33]), supporting H4b.

The serial mediation model (Hypothesis 5) was tested using Hayes’ PROCESS macro (Model 6) with 2,000 bootstrap resamples and bias-corrected 95% confidence intervals (83). The total association between expectation confirmation and continuance intention was significant (*B* = 0.75, *t* = 14.97, 95% CI [0.65, 0.85], *p* < 0.001). None of the control variables was significantly associated with continuance intention, including gender (*B* = −0.09, *p* > 0.05), age (*B* = 0.04, *p* > 0.05), education level (*B* = 0.09, *p* > 0.05), and monthly income (*B* = −0.04, *p* > 0.05). Expectation confirmation was significantly associated with perceived performance (*B* = 0.53, SE = 0.04, t = 12.19, 95% CI [0.44, 0.61], *p* < 0.001). Perceived performance was significantly associated with satisfaction (*B* = 0.34, SE = 0.05, *t* = 6.91, 95% CI [0.25, 0.45], *p* < 0.001), and satisfaction was significantly associated with continuance intention (*B* = 0.33, SE = 0.06, *t* = 5.11, 95% CI [0.20, 0.46], *p* < 0.001). The serial indirect association was significant (*B* = 0.06, BootSE = 0.02, 95% CI [0.03, 0.11]), providing support for Hypothesis 5. Given the cross-sectional design of the current study, these findings indicate an indirect association that is statistically consistent with the proposed sequential pathway but do not establish causal relationships among the key constructs. Figure 2 presents the results of the hypothesized model.

**Figure 2:**
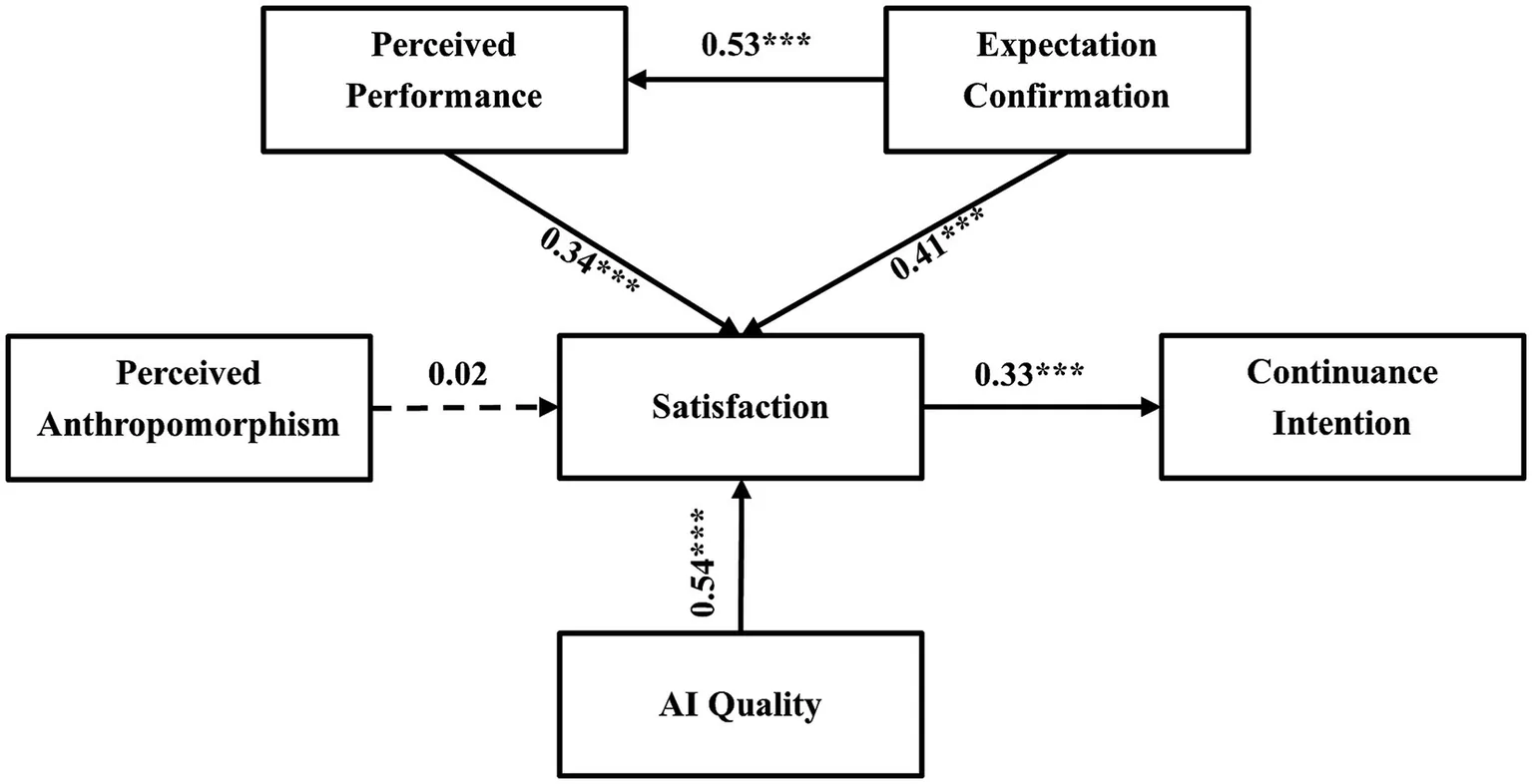
Results of the model of antecedents of young Chinese adults’ continuance intention toward AI-driven mHealth apps. ***p < 0.001.

## 5 Discussion

The rapid development of AI-driven mHealth apps has generated substantial global impact and increasing scholarly attention toward individuals’ continuance intention toward these apps (10–12), particularly in China, where the AI health industry has expanded rapidly within a collectivist cultural context. Guided by ECM, this study incorporates established theoretical constructs and AI-related features to examine their associations with continuance intention. Using a cross-sectional online survey design, the study examined hypothesized associations, including mediation and serial mediation, among young Chinese adults aged 18–34 years. The findings provide empirical insights into the explanatory value of ECM in the emerging AI-driven mHealth context and offer practical implications for promoting sustained engagement with AI-driven mHealth apps.

As previous empirical findings have suggested, expectation confirmation and perceived performance are key components that are positively associated with satisfaction (42–44, 46, 47). For instance, research in the digital health domain indicated that the greater users’ evaluations that healthcare platforms meet their initial expectations, the higher their level of satisfaction (44). Moreover, perceived performance has been found to be positively associated with satisfaction (46). Consistent with prior studies, the current study showed a comparable pattern, whereby expectation confirmation and perceived performance were positively associated with satisfaction with AI-driven mHealth apps among young Chinese adults. In terms of the direct associations between AI-related features and satisfaction, the present study found that AI quality was positively associated with user satisfaction with AI-driven mHealth apps. This finding is consistent with prior evidence suggesting a positive association between perceived quality and consumer satisfaction with mHealth and e-government services and related platforms (84–86). However, the finding regarding perceived anthropomorphism differed from prior research. While recent evidence has indicated that perceived anthropomorphism is positively associated with users’ satisfaction with AI humanoid service bots (55, 57), the current study did not find evidence of a positive association between perceived anthropomorphism and users’ satisfaction with AI-driven mHealth apps (B = 0.02, t = 0.83, 95% CI [−0.03, 0.08], *p* > 0.05). One possible explanation is that users may assume that AI-driven platforms can only simulate emotional interactions and cannot fully reproduce human–human connections in healthcare settings (87).

To evaluate the mediation hypotheses (H3a–b), mediation analysis was conducted. First, a significant indirect association through satisfaction was observed between the main Expectation Confirmation Model (ECM) constructs (expectation confirmation and perceived performance) and continuance intention among young Chinese adults. This aligns with previous empirical research indicating that expectation confirmation is positively associated with satisfaction (42–44). Additionally, higher levels of perceived performance were associated with greater satisfaction (46, 47). In turn, individuals’ satisfaction with mHealth apps was positively linked to their continuance intention, a relationship consistent with observations specifically within the domain of health management apps (35, 62). Regarding the AI-related features, the mediating role of user satisfaction between these features and continuance intention (H4a–b) was partially supported. Specifically, the results demonstrated a positive indirect pathway from AI quality to continuance intention through user satisfaction. This finding is consistent with prior evidence suggesting a positive association between perceived quality and consumer satisfaction across online-to-offline commerce, tourism services, and AI-enabled e-banking contexts (59–61), with consumer satisfaction also positively associated with users’ continuance intentions (62).

Conversely, satisfaction did not emerge as a significant mediator in the relationship between perceived anthropomorphism and continuance intention. This finding diverges from prior literature reporting a positive indirect association along this pathway (35, 50, 54). This difference may indicate that the association between perceived anthropomorphism and continuance intention is not adequately captured by satisfaction alone, suggesting that other mediating mechanisms may warrant consideration. For instance, prior research has demonstrated a positive association between perceived anthropomorphism and perceived AI empathy (88). In other words, when users perceive greater human-like characteristics in AI applications during their interactions, they may also perceive the application as more capable of understanding, recognizing, and responding to their emotional states or needs. Furthermore, previous research among customers using tourism chatbots in China has demonstrated a positive association between perceived AI empathy and user satisfaction (89). Taken together, these findings point to a potential serial mediation pathway involving perceived anthropomorphism, AI empathy, satisfaction, and continuance intention, which could be examined in future research.

Lastly, the serial mediation hypothesis was supported, indicating a significant indirect association between expectation confirmation and continuance intention that was statistically consistent with the proposed sequential pathway through perceived performance and satisfaction. This finding is consistent with previous literature indicating that expectation confirmation is positively associated with perceived performance (41, 64). Moreover, higher perceived performance tends to correspond with greater levels of satisfaction (42, 47), which, in turn, is positively related to continuance intention (35, 62). Overall, the current findings shed light on the mechanisms beyond the direct association pathway of expectation confirmation and continuance intention within the domain of AI-driven mHealth apps among young Chinese adults, highlighting the psychological factors associated with their continuance intention.

The findings of this study offer theoretical insights into the existing literature on digital health technology adoption. First, this study demonstrates that young Chinese adults’ continuance intentions toward AI-driven mHealth apps are largely aligned with the framework of the Expectation Confirmation Model. Specifically, the core constructs of expectation confirmation and perceived performance are strongly associated with user satisfaction, which is also positively associated with continuance intention. Consequently, this study offers support for the explanatory power of ECM within the specific domain of AI-driven healthcare platforms, particularly when applied to a younger demographic within a collectivistic cultural context like China. Second, the current study provides partial support for the CASA perspective and extends prior research by showing that AI quality can represent a parallel correlate of satisfaction alongside the core ECM constructs (48–50). This extends the application of ECM by suggesting that model extensions do not necessarily require new constructs to be positioned as antecedents within the ECM evaluation chain but may also be conceptualized as parallel correlates of satisfaction. Third, perceived anthropomorphism was not significantly associated with satisfaction, although prior studies have found support for this relationship. This inconsistent finding provides new insight into ECM in the context of AI-specific health management apps. One possible explanation is that AI-driven platforms can simulate emotional interactions but cannot fully reproduce human–human connections in healthcare settings. Future studies should consider different perspectives and AI-specific constructs when extending ECM to further enhance its theoretical applicability in AI-driven health management apps.

From a practical perspective, the findings have twofold implications. First, the results indicate that expectation confirmation and perceived performance are positively associated with satisfaction among young Chinese adults, and satisfaction is positively associated with continuance intention. To support sustained use, developers should prioritize the technical reliability and performance of AI-driven health management and monitoring functions to better align with user needs. Consistent with this, although prior literature highlights the potential of AI-based healthcare platforms, particularly in deep learning and early disease detection in mHealth (90), empirical evidence in this specific context remains emerging, thereby underscoring the need for further validation of how such capabilities translate into user outcomes such as satisfaction and continuance intention. Second, a recent systematic review indicated that acceptance of AI in health care varies across users, with barriers including trust in AI applications and reduced interpersonal interaction, while AI-related knowledge and education were identified as important factors in promoting acceptance (91). Accordingly, multimedia educational videos may provide a useful strategy for increasing users’ awareness and knowledge of AI technologies (91). Given the role of short-form video platforms such as Douyin in disseminating health information in China (92), collaboration between health experts and industry practitioners to create content promoting AI-driven mHealth apps may help narrow the technology diffusion gap and support sustained use.

Several limitations of this study should be acknowledged. First, respondents may have had different experiences with AI-driven mHealth apps. Although all respondents had prior experience with such applications, including hospital diagnostic services, health monitoring tools, or AI-based health chatbots, differences in app use may have shaped their perceptions of anthropomorphism, perceived performance, and other app characteristics. Future studies are recommended to focus on a specific type of AI-driven mHealth apps to provide a more consistent referent and further explore users’ continuance intention. Second, this study used a cross-sectional design. Although the mediation and serial mediation analyses identified statistically significant indirect associations consistent with the proposed theoretical pathways, the cross-sectional design limits the ability to establish temporal ordering or causal relationships among the constructs. Future studies could employ longitudinal designs to further examine these relationships. Third, the use of non-probability sampling, together with the sample’s relatively high educational attainment and income level (52.9% earning above CNY 8,000 per month), may limit the generalizability of the findings to young Chinese adults with different socioeconomic backgrounds. Furthermore, non-response bias could not be directly examined because information on non-respondents was unavailable. Future studies should employ more diverse and representative sampling strategies and assess potential non-response bias. Fourth, this study did not account for moderating variables, which may have obscured interaction effects. Therefore, researchers and academic experts are encouraged to further explore factors such as health information privacy concerns, AI literacy, and perceived health stigma to contribute to a more comprehensive understanding of the conditions under which AI-driven mHealth apps are associated with sustained user engagement. Fifth, while descriptive studies have indicated differences between frequent or heavy and lighter users of AI-driven mHealth apps, the current study focused on explanatory relationships and did not examine potential differences among respondents with different levels of use. Future studies should incorporate usage-frequency or intensity measures to further examine such differences. Lastly, satisfaction and continuance intention were measured using only two items, while AI quality was measured using four items as a single reflective construct. Although these measures met relevant reliability and validity thresholds, the limited number of items may not fully capture these constructs. Future research could use more comprehensive measures to provide broader coverage.

## 6 Conclusion

As emerging artificial intelligence technologies continue to reshape the healthcare industry, AI-driven mHealth apps have garnered significant scholarly attention (10–12). However, empirical understanding of how AI-specific features are associated with users’ continued usage intentions remains limited. To address this gap, this study investigates young Chinese adults by applying an extended Expectation Confirmation Model integrated with key AI-related feature constructs. This framework allows for an examination of the direct, mediated, and serial mediation associations among the proposed variables. The study contributes to the literature by providing empirical evidence on these associations within the context of AI-driven mHealth apps.

The findings highlight the importance of supporting continued use of AI-driven mHealth platforms by prioritizing technical reliability and high-performance AI capabilities. From a practical perspective, this study offers two key suggestions. First, health experts and developers should prioritize the technical reliability and performance of AI-driven mHealth apps to support user satisfaction and continued use. Second, health experts and industry practitioners should collaborate to develop educational content that enhances users’ awareness and knowledge of AI-driven mHealth technologies, particularly through short-form videos, which have gained increasing attention as a contemporary approach to health education.

The present study has several limitations that warrant consideration. First, the use of non-probability sampling limits the generalizability of the findings to young Chinese adults with different socioeconomic backgrounds. Second, non-response bias could not be assessed and should be considered in future research. Third, although this study examined mediation and serial mediation pathways, further research is needed to investigate additional factors that may explain the associations between the extended ECM constructs and continuance intention. Finally, although satisfaction and continuance intention demonstrated acceptable reliability and validity, the use of relatively limited measurement items may not fully capture all dimensions of these constructs. Future research could therefore consider more comprehensive measures.

## Data Availability

The original contributions presented in the study are included in the article/supplementary material, further inquiries can be directed to the corresponding author/s.
